# The Role of Hyperbaric Oxygen Therapy in Management of Necrotizing Soft Tissue Infection

**DOI:** 10.3390/jcm14103511

**Published:** 2025-05-17

**Authors:** Thomas J. Gregory, Kinjal Sethuraman

**Affiliations:** R Adams Cowley Shock Trauma Center, University of Maryland School of Medicine, Baltimore, MD 21201, USA; ksethuraman@som.umaryland.edu

**Keywords:** hyperbaric oxygen, necrotizing infection, reactive oxygen species, inflammation, gas gangrene, alpha toxin

## Abstract

Necrotizing soft tissue infection (NSTI) is a life-threatening, high morbidity pathology that requires aggressive, multidisciplinary management. Surgery and antibiotic administration are core components of treatment. Adjunctive incorporation of hyperbaric oxygen therapy (HBOT) can further enhance treatment and recovery. Benefit is achieved through multiple effects brought about by increase of local and systemic oxygen tension. Direct effects include bacteriostasis, disruption of bacterial toxin production, and attenuation of inflammation. Indirect benefits include demarcation of viable tissue to enhance surgical efforts, potentiation of antibiotics, and enhancement of immune system function. Overall, HBOT has few contraindications and is typically well tolerated by patients. Treatment course and appropriateness of individual patients can be determined through consultation with Hyperbaric Medicine specialists. The benefits of HBOT in morbidity and mortality of NSTI have been well demonstrated and this therapy should be considered as a component of care to all affected patients.

## 1. History

Necrotizing soft tissue infection (NSTI) is a devastating disease with significant morbidity and mortality [1]. Care for afflicted patients involves a multidisciplinary approach and a combination of therapeutic interventions. Hyperbaric Oxygen Therapy (HBOT) has been in use for decades and is applied in the treatment of many indications, summarized in Table 1 [2,3,4].

The advantageous effects of HBOT in soft tissue infection were demonstrated as early as 1960 [5]. Continued study over the decades has shown decreased morbidity and mortality in NSTI patients whose care included HBOT [6,7,8].

## 2. Mechanisms of Action

To explain the mechanisms of HBOT is to follow principles of physics into clinical application. Boyle’s Law describes the relationship between pressure and gas volume, seen in the shrinking of gas spaces such as air emboli or other bubbles in the pressurized environment of the hyperbaric chamber. Conversely, a gas space filled under presssure, if unable to vent, will increase in volume upon removal from pressure [9].

**Table 1:** Hyperbaric Oxygen Therapy Applications.

Current UHMS Approved Indications	Emerging Indications
Gas embolism	Traumatic Brain Injury
Decompression Sickness	Long Covid
Carbon Monoxide Toxicity	Calciphylaxis
Necrotizing Soft Tissue Infection	Frostbite
Compromised Surgical Flaps and Grafts	Raynaud’s/Systemic Sclerosis Ulcers
Sudden Sensorineural Hearing Loss	Pyoderma Gangrenosum
Intracranial Abscess	Inflammatory Bowel Disease ● Crohn’s Disease ● Ulcerative Colitis ● Pouchitis
Chronic Refractory Osteomyelitis	
Severe Anemia	
Avascular Necrosis	
Diabetic Foot Ulcer	
Acute Ischemias ● Central Retinal Artery Occlusion ● Crush Injury ● Compartment Syndrome ● Burns	
Delayed Radiation Injury ● Soft Tissue Radionecrosis ● Osteoradionecrosis ● Radiation Cystitis ● Radiation Proctitis	

Tabulated from the Undersea and Hyperbaric Medical Society (UHMS) Hyperbaric Medicine Indications Manual [3,4].

Increased dissolution of molecules at increased pressure is described by Henry’s Law [9]. Pathology develops from this principle in the opposite direction, with gases dissolved under pressure coming out of solution as pressure decreases, forming bubbles with a variety of detrimental effects. This is the fundamental mechanism of decompression sickness, which affects divers or compressed air workers. The principle of Henry’s Law also underlies the increased content of oxygen dissolved in the blood during HBOT.

Through Dalton’s Law of Partial Pressures, one can understand the fundamental increase in oxygen delivery under elevated atmospheric pressure compared to simply increasing the fraction of inspired oxygen (F_I_O_2_) in a normobaric setting. This law explains that the total pressure of a gas mixture, such as regular room air, can be understood as a total of partial pressures of the component gases. The partial pressure of a gas is simply its component fraction multiplied by the pressure of the whole mixture [9]. For example, a SCUBA diver breathing from a tank of regular air is breathing 78% nitrogen—normal air composition. The mechanical components of SCUBA equipment deliver air from the supply tank at a pressure equal to ambient pressure. While floating on the surface at standard sea level conditions, the diver is at 1.0 atmosphere absolute (ATA) of pressure, as is the gas delivered from the air tank. In this setting, the partial pressure of nitrogen delivered is 0.78 ATA—78% of the 1.0 total. As the diver descends, ambient pressure increases. At a depth where the added pressure of water contributes an additional atmosphere of pressure, the total is 2.0 ATA. Breathing here, the same gas mixture breathed on the surface now delivers 1.56 ATA of nitrogen to the diver—78% of the total. This component 1.56 ATA of the total 2.0 ATA is the partial pressure of nitrogen per Dalton’s Law.

The impact of HBOT on NSTI is a result of multiple changes induced by increased oxygen tension. Such a physiologic effect is achieved through a patient breathing 100% oxygen gas, a near five-fold increase to normal air composition of 21% oxygen. This is seen at the F_I_O_2_ variable of the alveolar gas equation:

$$PAO_{2}=F_{I}O_{2}\;\left(Patm-PH_{2}O\right)-PCO_{2}\;\left[F_{I}O_{2}+\frac{1-F_{I}O_{2}}{RER}\right]$$

While receiving maximized F_I_O_2_, the patient is concurrently pressurized in a hyperbaric chamber to greater than the normal ambient one atmosphere of pressure, typically 2.0 to 3.0 ATA. While less emphasized in most clinical settings, the hyperbaric treatment environment demands attention to the athospheric pressure variable of the equation (Patm). Through the lens of Dalton’s Law of Partial Pressures, room air breathing at standard sea level pressure yields 0.21 ATA of oxygen. Maximized F_I_O_2_ of 100% increases this to 1.0 ATA of oxygen. A hyperbaric treatment profile of 2.0 ATA pressure doubles this to a delivery of 2.0 ATA pressure of oxygen to the body. This combination of maximal oxygen inhalation under elevated pressure results in a patient receiving approximately 10 to 15 times greater oxygen than normal breathing.

The increased oxygenation occurs local to the wound and systemically, both to therapeutic result. The impacts of this include first order effects as well as indirect benefits through enhancement of innate mechanisms and concurrent therapies, as summarized in Table 2. In all forms of NSTI, the effects of HBOT include bacteriostasis and boosts to immune system functions, anti-inflammatory action, and antibiotic potentiation. In the NSTI subset of gas gangrene, there is the additional benefit of stagnation of Clostridial alpha toxin production.

## 3. Bacteriostasis

NSTI can result from infection by several organisms and is commonly polymicrobial on culture assessment. Among the commonly isolated organisms are obligate and facultative anaerobic bacteria [10]. These organisms, and some fungal pathogens, can cause, and in turn thrive and spread in, a hypoxic environment [11].

By markedly increasing tissue oxygen levels through HBOT, a swell occurs in production of reactive oxygen species (ROS) [12]. The oxidative stress produced by this alteration to the tissue environment has a toxic effect on multiple components of bacterial cells, including DNA, RNA, lipid bilayer, and proteins. Impact from ROS damages both nucleic acid chains and free nucleotides. Damage to lipid molecules impairs their function within bacterial structures, including the cell membrane. Bacterial lipid peroxidation can occur, causing effects similar to native immune cell attack during phagocytosis [13]. Peptide molecules with various functions in bacteria are altered through oxidative alteration. The cumulative result of these changes is an overall bacteriostatic effect.

## 4. Antibiotic Potentiation

Along with surgical debridement, antibiotic therapy remains a core component of NSTI treatment. In addition to more direct effects, HBOT can have an enhancing effect on antibiotics.

In a 2009 study assessing treatment of experimental mediastinitis in rats, HBOT in combination with antibiotics was found to cause a greater reduction in methicillin resistant Staphylococcus aureus (MRSA) bacterial counts when compared to antibiotics or HBOT alone. This study found the improved combination effect on all three medications assessed: linezolid, vancomycin, and teicoplanin [14]. Similar antibiotic potentiation has been found in cefazolin against rat model osteomyelitis from *S. aureus* [15], in ciprofloxacin targeting experimental Pseudomonas biofilms [16], and in tobramycin therapy for rat model *S. aureus* infective endocarditis [17].

Investigation into the molecular mechanisms of antibiotic efficacy have indicated that killing effects are often achieved through an increased production of ROS. Under anaerobic conditions, the antibiotic killing effect is impaired. Conversely, it is enhanced by increased oxygen exposure [18]. In a 2012 study of antibiotic efficacy against Mycobacterium colonies, a subset of bacteria within larger colonies sometimes survived the initial wave of killing. This “persister” survival was found to be due to decreased concentration of ROS from dissolved oxygen depletion. In turn, an increase of the dissolved oxygen achieved complete sterilization of the bacteria colonies [19].

This impact of adequate oxygenation for antibiotic efficacy is seen in multiple antibiotics at the cellular level of action. Fluoroquinolone and Beta-lactam antibiotics exert their bacteriocidal effects via ROS production [20]. Aminoglycosides reach their target via oxygen dependent cellular transport mechanisms across the bacteria cell membrane [21]. Pseudomonas aeruginosa can increase aminoglycoside resistance via a shift to anaerobic metabolism. In a hyperbaric oxygen environment, aminoglycoside sensitivity increases as these same bacteria are induced to shift into aerobic metabolism [20].

## 5. Immune System Enhancement

NSTI treatment mainstays include assertive action of surgical debridement and targeting of infectious organisms through antibiotic therapy. In the background, as with all infections, the cure is ultimately dependent on host immune mechanisms. Any active infection increases metabolic demand. NSTI can further deprive tissues of oxygen through ischemic effects of tissue inflammation and damage. This oxygen depletion inhibits the efficacy of the immune system.

The variety of immune cells is broad and the relationship of cellular actions and signaling is intricate. Well-described by Yan et al., polymorphonuclear neutrophils (PMN) are the most abundant among our innate immune cells and serve as a primary mediator in fighting infection [22]. There are several mechanisms by which PMNs exert antimicrobial effects. Many of these, including ROS production and organism iodination, are dependent upon oxygen presence [23]. In some bacteria, the destructive effect of PMN phagocytosis is impaired by hypoxia, while it has been shown to be enhanced by HBOT [24].

It bears acknowledgement that PMNs are also capable of antimicrobial mechanisms of action under an anaerobic metabolic state [23]. These, of course, are not reliant upon or enhanced by HBOT. However, no NSTI clinical course involves a permanent stay in the hyperbaric chamber, so those aspects of immune response less dependent on oxygenation continue regardless of HBOT incorporation.

There are a variety of ROS involved in the actions of PMNs. Myeloperoxidase (MPO) and superoxide dismutase (SOD) are two key components, which have been studied by Hedetoft et al. for their role in NSTI. They found that both of these molecules increased in patients after HBOT [25].

The same study found an increase in heme-oxygenase-1 (HO1) in NSTI patients after HBOT. HO1 products can enhance the phagocytosis of bacteria by immune cells and a decreased level of HO1 is associated with higher mortality in sepsis [26].

## 6. Inflammation Attenuation

For any health care provider who has cared for a patient in the post-operative period or during an infection, the challenge of inflammation in managing pain, wound-healing, and overall therapeutic goal is familiar. Amelioration of inflammation is a significant benefit of HBOT.

The role of host PMNs is crucial in initial stages of combating infection, though their effects are not purely positive overall. Chemotaxis to an infected space and ensuing antimicrobial effects are crucial to bacterial killing but later lead to inflammation and local tissue damage [22,27]. While a ubiquitous and potent mediator of infection, the PMN has poor ability to differentiate between invasive microbes and host tissue. Previously discussed ROS, along with antimicrobial peptides and proteases, have been shown to cause host tissue damage in the inflammatory stages of wound response [28].

After initial recruitment to a region of infection, local PMN adhesion is facilitated through integrin molecules. Research has shown a decrease in PMN integrin expression in subjects receiving HBOT compared to controls [29]. In the same study, pro-inflammatory immune markers, namely TNF-α and IL-1β cytokines, were present in lower levels in the HBOT recipients.

Another molecule of interest in inflammatory states is heme-oxygenase-1 (HO1). Beyond having an antimicrobial effect, it has been found that HO1 plays a role in attenuating the inflammatory response [30]. A higher level of HO1 is a notable consideration in the anti-inflammatory role of HBOT [25].

Detrimental effects of impaired tissue perfusion are extensive, with improved bacterial growth, decreased antibiotic delivery, and immune impairment already discussed. Notably, the initiation of the inflammatory cascade involves signals stimulated by oxygen deprivation [31]. During HBOT, the uniquely oxygen-deprived environment of NSTI receives greatly increased perfusion. A profound effect of HBOT is the ability to perfuse tissue from oxygen dissolved directly into plasma, not just that carried by hemoglobin [32]. Part of the impairment to perfusion in NSTI is a result of deformation of tissue and associated blood vessels. This can impair the flow of red blood cells (RBCs) through microcirculation, depriving the tissue of oxygen delivery. Movement of RBCs through capillaries is dependent on cell membrane deformability, which is unique to the RBC [33]. HBOT has been shown to increase the degree of RBC deformability [34]. This, having somewhat longer duration of effect than direct plasma oxygen dissolution, serves to further improve the affected tissue perfusion and alleviate the harmful milieu of ischemia.

## 7. Gas Gangrene Toxin Degradation

A subtype of NSTI is gas gangrene, also referred to as Clostridial myonecrosis or Clostridial myositis [35]. This aggressive pathology, characterized by extensive tissue damage and gas production, is a result of infection from bacteria of the genus Clostridium. There are numerous species within the genus. *C. perfringens* and *C. septicum* are among the most commonly isolated, with *C. perfingens* commonly associated with post-traumatic gas gangrene and *C. septicum* with non-traumatic gas gangrene [36].

Clostridial species are anaerobic and sensitive to elevated levels of oxygen. Under HBOT, tissue oxygen tension levels can be reached that are bacteriostatic to these organisms [37].

While colonization and expansion of the Clostridial colony itself is of concern in gas gangrene, the uniquely detrimental aspect of these infections is the production of exotoxins. Alpha toxin is the most pertinent of the many produced, causing destruction and impairment to multiple tissues and cell types, including platelets, immune cells, and capillaries [38,39]. Toxin production and infection progression occurs rapidly and can expand faster than the host can develop immune mechanisms to combat it [40].

In addition to the bacteriostatic effect against the Clostridia bacterium directly, elevated oxygen tension in tissues from HBOT also halts production of alpha-toxin [37,41]. There has not been a demonstrated effect of HBOT on existing toxins [42]. However, a temporary break in the rapid cascade of toxin expansion can allow the host immune system to begin detoxification of these molecules.

As with all NSTI, surgical debridement and washout and antibiotic therapy remain the mainstays of gas gangrene treatment. It has been indicated through numerous studies that mortality and morbidity decrease with the addition of HBOT to this multidisciplinary regimen [37,43].

## 8. Incorporation of HBOT

The primary management of any NSTI lies in the combination of surgical intervention and antibiotic therapy. Indeed, many health systems lack the option to incorporate HBOT into the NSTI patient course. Where possible, however, there are extensive data (Table 3) from experimental studies, animal trials, and human patient case collectives to support incorporation of this therapy into the overall multidisciplinary course [44].

HBOT can augment and complement the action of multiple antibiotics [12]. The increased tissue perfusion can also optimize surgical management through improved demarcation of viable tissue and possible reduction of surgery extent through stasis of bacteria and toxins [12,37]. HBOT has, in fact, been associated with decreased amputations and improved survival in NSTI in a retrospective cohort study from 2004. The odds ratio of survival was found to be 8.9 in this cohort, favoring the population receiving HBOT [45]. A 2021 meta-analysis of over 47,000 NSTI patients further supported these associations [46].

In a review of cases from fourteen institutions over a three-year period, NSTI patients with high illness severity showed increased survival when treated with HBOT compared to controls, with death rates of 4% versus 23%, respectively [6]. Many institutions experienced interruption to normal clinical operations, including HBOT, during the initial response to COVID-19. During these first several months, a single institution where HBOT is standard practice for NSTI had an increased number of patients who did not receive this treatment. In comparison to the NSTI patient groups treated with and without HBOT over a 36-month study period, approximately 56% received HBOT. Only 20% of patients received HBOT during the period of greatest COVID-19 restrictions, down from 68% prior to that. Comparison showed 15.4% mortality in control patients, while the rate was 5.8% in those who received HBOT. Those patients with high illness severity by APACHE II score or with larger wounds had lower risk of death when treated with HBOT. Those with greater illness severity and larger wounds showed the greatest decrease in death risk, with a 0.23 odds ratio [47].

Hyperbaric Medicine remains a small field relative to other medical specialties, with variability in practice between centers. Indeed, among the literature studying the efficacy of HBOT in NSTI, there is variation among the treatment profiles used in regard to dosing factors of pressure and time. Outside of literature reviews and experimental settings, the realities of practice, such as chamber type, staffing limitations, and patient characteristics, can affect the specifics of HBOT utilization. With these factors noted, there are recommended regimens for NSTI. The following guidance on an HBOT regimen is provided in the Undersea and Hyperbaric Medical Society Hyperbaric Medicine Indications Manual [48]:

### 8.1. Recommended HBOT Regimens

#### 8.1.1. General NSTI [48]

- 2.0–2.5 ATA pressure
- 90 min of oxygen delivery
- Twice daily for several days, until apparent infection control and halt of necrosis
- Once daily after stabilization to avoid relapse until persistent control is apparent

#### 8.1.2. In the Subset of Gas Gangrene NSTI [48,49]

- 2.8–3.0 ATA pressure
- 90 min of oxygen delivery
- 2 to 3 times in first 24 h
- Twice daily for following 2 to 5 days
- Extend treatment while patient remains toxic
- Utilization review after 10 treatments

Due to center variability and unique demands and restrictions, a regimen as robust as these may not be possible in many cases. Prudence dictates a similar goal, with emphasis on the underlying guidelines of early HBOT application and consistent use until apparent infection control.

As with any medical therapy, HBOT carries benefits and risks (Table 4). Overall, complications are rare, and side effects are mild or easily mitigated [50]. Some potential side effects are seen more frequently in patients with HBOT indications requiring very long treatment courses. These are not typical of the expected course for NSTI. Patients should be evaluated by physicians with expertise in Hyperbaric Medicine with informed consent obtained from patients or authorized representatives after risk and benefit discussion.

There are few contraindications to prevent a patient from undergoing HBOT. The only absolute contraindications include untreated pneumothorax, which could worsen under the pressure dynamics of treatment; any state that would pose a threat to patient or staff safety, such as altered mental status with combativeness; and implanted devices that are not pressure-compatible and critical to keep in place, such as some older pacemakers.

Relative contraindications to HBOT, which can typically be resolved with consideration to timing or prophylactic therapies, include certain chemotherapy regimens, anxiety, claustrophobia, hypoglycemia, hemodynamic instability, and intolerance to pressure changes.

Contraindicated chemotherapy is a simple incompatibility resolved with delay between dosing and start of HBOT. Anxiety, claustrophobia, hypoglycemia, and sometimes intolerance to pressure changes may be resolved with pre-treatment medication or blood glucose correction.

Hemodynamic instability as a contraindication will be dependent on the capabilities of the individual hyperbaric facility. Several institutions are capable of carrying out HBOT on critically ill patients while on pressor medications, ventilators, or cerebral monitoring devices. Such a capacity should not, however, be considered the norm and all patients should be discussed with the Hyperbaric Medicine service to determine capability. Any case of severe instability, requiring active or very frequent cardiac resuscitation, should be held from HBOT until more stable. While CPR and other ACLS measures can be carried out in a few capable centers, in none is it an ideal setting and serious pause should be given before continuing HBOT if severe decompensation is likely.

Intolerance to pressure change is variable from patient to patient. It can arise from discomfort in the gas spaces of the body, namely the gut, lungs, sinuses, and middle ear. The most frequent issue among these, by far, is with pressure equalization of the middle ear. Inadequate equalization can result in barotrauma to the tympanic membrane, with pain and hearing loss potentially resulting. In alert patients, this can often be avoided through coaching on equalization techniques. Medications such as intranasal oxymetazoline are often used with anecdotal success, but less supportive data occur in some studies [55]. For those patients who can not accomplish ear equalization, whose progress with HBOT is impeded by ear pain, venting of the middle ear is an option via tympanic membrane myringotomy or tympanostomy tube placement. These procedural options are of significant consideration in NSTI, as many patients are critically ill and sedated or remain intubated between planned surgeries. Such a state, of course, precludes patient participation in ear equalization and raises their risk of ear barotrauma. As with other potential contraindications, the individual patient scenario should be evaluated by the Hyperbaric Medicine practitioner and appropriate measures taken to facilitate successful HBOT.

## 9. Summary

In the approach to the patient afflicted by NSTI, multidisciplinary care is the standard. Aggressive surgical intervention and antibiosis are the mainstays of treatment, but hyperbaric oxygen therapy offers a helpful adjunct. The benefits seen in practice and literature include boosts to immune system and antibiotic function, attenuation of tissue inflammation, bacteriostasis, demarcation of viable tissue, and disruption of bacterial toxin production. The therapy is generally well tolerated and there are few contraindications. Alongside consultation with Hyperbaric Medicine providers for specialist insight, incorporation of hyperbaric oxygen therapy should be considered in management of all necrotizing soft tissue infections.

## Figures and Tables

**Table 2:** Mechanisms of HBOT in Treatment of NSTI.

Bacteriostasis
Antibiotic potentiation
Immune system enhancement
Inflammation attenuation
Alpha toxin disruption

**Table 3:** Literature Summary of HBOT Benefits in NSTI Care *.

Author, Date	Findings
Shaw JJ et al., 2014 [6]	Reduced mortality, decreased complications
Devaney B et al., 2015 [7]	Reduced mortality
Mladenov A et al., 2022 [8]	Reduced mortality
Turhan V et al., 2009 [14]	Linezolid, vancomycin, and teicoplanin potentiation
Mendel V et al., 1999 [15]	Cefazolin potentiation
Kolpen M et al., 2016 [16]	Ciprofloxacin potentiation
Lerche CJ et al., 2017 [17]	Tobramycin potentiation
Hedetoft M et al., 2021 [25]	Greater ROS presence
Baiula M et al., 2020 [29]	Decreased pro-inflammatory markers
Ryter SW; 2020 [30]	Increased inflammation attenuating molecules
Steenebruggen F et al., 2023 [34]	Greater RBC deformability
Bakker DJ; 2012 [37]	Improved morbidity, reduced mortality
van Unnik AJM; 1965 [41]	Impaired alpha toxin production
Hirn M; 1993 [43]	Improved morbidity, reduced mortality
Wilkinson D, Doolette D; 2004 [45]	Reduced mortality, decreased amputation
Hedetoft M et al., 2021 [46]	Reduced mortality, decreased amputation
Shishido A et al., 2025 [47]	Reduced mortality

* Selected publications only. Full bibliography in References section. Articles obtained via PubMed search and bibliography review of various hyperbaric medicine texts.

**Table 4:** Adverse effects associated with HBOT *.

Effect	Frequency
Ear pain	17%
Otic/sinus barotrauma	0.37%
Confinement anxiety	0.16%
Hypoglycemia	0.08%
Shortness of breath	0.05%
Seizure	0.02%
CHF exacerbation	2 in 906 treatments ** [51]
Pneumothorax	1 in 1.5 million treatments [52]
Progressive myopia (irreversible)	*** [53]
Accelerated cataract formation	***
Fire/explosion	Very rare, life-threatening [54]

Percentages are incidents per treatment. * Table adapted from: Sethuraman KN, Smolin R, Henry S. [50]. ** 2 CHF-like incidents out of cohort of CHF patients with a total of 906 HBOT sessions. One affected patient did not return after [51]. *** Associations seen in very long treatment courses not typical of standard care [53].

## Abbreviations

The following abbreviations are used in this manuscript:

NSTI	Necrotizing Soft Tissue Infection
HBOT	Hyperbaric Oxygen Therapy
PMN	Polymorphonuclear Neutrophil
RBC	Red Blood Cell
CPR	Cardiopulmonary Resuscitation
ACLS	Advance Cardiovascular Life Support
ATA	Atmosphere Absolute
ROS	Reactive Oxygen Species

## References

[1] Madsen M.B., Skrede S., Perner A., Arnell P., Nekludov M., Bruun T., Karlsson Y., Hansen M.B., Polzik P., Hedetoft M. (2019). Patient’s characteristics and outcomes in necrotising soft-tissue infections: Results from a Scandinavian, multicentre, prospective cohort study. Intensiv. Care Med..

[2] Smolle C., Lindenmann J., Kamolz L., Smolle-Juettner F.M. (2021). The History and Development of Hyperbaric Oxygenation (HBO) in Thermal Burn Injury. Medicina.

[3] Huang E. (2023). UHMS Hyperbaric Medicine Indications Manual.

[4] Bennett M.H., Mitchell S.J., Buckey J.C., Robins M. (2023). Emerging Indications. Hyperbaric Medicine Indications Manual.

[5] Brummelkamp W.H. (1965). Considerations on hyperbaric oxygen therapy at three atmospheres absolute for clostridial infections type welchii. Ann. N. Y. Acad. Sci..

[6] Shaw J.J., Psoinos C., Emhoff T.A., Shah S.A., Santry H.P. (2014). Not just full of hot air: Hyperbaric oxygen therapy increases survival in cases of necrotizing soft tissue infections. Surg. Infect..

[7] Devaney B., Frawley G., Frawley L., Pilcher D.V. (2015). Necrotising soft tissue infections: The effect of hyperbaric oxygen on mortality. Anaesth. Intensive Care.

[8] Mladenov A., Diehl K., Müller O., von Heymann C., Kopp S., Peitsch W.K. (2022). Outcome of necrotizing fasciitis and Fournier’s gangrene with and without hyperbaric oxygen therapy: A retrospective analysis over 10 years. World J. Emerg. Surg..

[9] Hardy K., Neuman T.S., Thom S.R. (2008). Physics of Hyperbaric Oxygen Therapy in Physiology and Medicine of Hyperbaric Oxygen Therapy E-Book.

[10] Hakkarainen T.W., Kopari N.M., Pham T.N., Evans H.L. (2014). Necrotizing soft tissue infections: Review and current concepts in treatment, systems of care, and outcomes. Curr. Probl. Surg..

[11] André A.C., Laborde M., Marteyn B.S. (2022). The battle for oxygen during bacterial and fungal infections. Trends Microbiol..

[12] Memar M.Y., Yekani M., Alizadeh N., Baghi H.B. (2019). Hyperbaric oxygen therapy: Antimicrobial mechanisms and clinical application for infections. Biomed. Pharmacother..

[13] Fang F.C. (2004). Antimicrobial reactive oxygen and nitrogen species: Concepts and controversies. Nat. Rev. Microbiol..

[14] Turhan V., Sacar S., Uzun G., Sacar M., Yildiz S., Ceran N., Gorur R., Oncul O. (2009). Hyperbaric oxygen as adjunctive therapy in experimental mediastinitis. J. Surg. Res..

[15] Mendel V., Reichert B., Simanowski H.J., Scholz H.C. (1999). Therapy with hyperbaric oxygen and cefazolin for experimental osteomyelitis due to Staphylococcus aureus in rats. Undersea Hyperb. Med..

[16] Kolpen M., Mousavi N., Sams T., Bjarnsholt T., Ciofu O., Moser C., Kühl M., Høiby N., Jensen P.Ø. (2016). Reinforcement of the bactericidal effect of ciprofloxacin on Pseudomonas aeruginosa biofilm by hyperbaric oxygen treatment. Int. J. Antimicrob. Agents.

[17] Lerche C.J., Christophersen L.J., Kolpen M., Nielsen P.R., Trøstrup H., Thomsen K., Hyldegaard O., Bundgaard H., Jensen P.Ø., Høiby N. (2017). Hyperbaric oxygen therapy augments tobramycin efficacy in experimental Staphylococcus aureus endocarditis. Int. J. Antimicrob. Agents.

[18] Dwyer D.J., Belenky P.A., Yang J.H., MacDonald I.C., Martell J.D., Takahashi N., Chan C.T., Lobritz M.A., Braff D., Schwarz E.G. (2014). Antibiotics induce redox-related physiological alterations as part of their lethality. Proc. Natl. Acad. Sci. USA.

[19] Grant S.S., Kaufmann B.B., Chand N.S., Haseley N., Hung D.T. (2012). Eradication of bacterial persisters with antibiotic-generated hydroxyl radicals. Proc. Natl. Acad. Sci. USA.

[20] Kok M., Maton L., van der Peet M., Hankemeier T., van Hasselt J.G.C. (2022). Unraveling antimicrobial resistance using metabolomics. Drug Discov. Today.

[21] Kapoor G., Saigal S., Elongavan A. (2017). Action and resistance mechanisms of antibiotics: A guide for clinicians. J. Anaesthesiol. Clin. Pharmacol..

[22] Yan J., Kloecker G., Fleming C., Bousamra M., Hansen R., Hu X., Ding C., Cai Y., Xiang D., Donninger H. (2014). Human polymorphonuclear neutrophils specifically recognize and kill cancerous cells. Oncoimmunology.

[23] Mandell G.L. (1974). Bactericidal activity of aerobic and anaerobic polymorphonuclear neutrophils. Infect. Immun..

[24] Almzaiel A.J., Billington R., Smerdon G., Moody A.J. (2013). Effects of hyperbaric oxygen treatment on antimicrobial function and apoptosis of differentiated HL-60 (neutrophil-like) cells. Life Sci..

[25] Hedetoft M., Jensen P.Ø., Moser C., Vinkel J., Hyldegaard O. (2021). Hyperbaric oxygen treatment impacts oxidative stress markers in patients with necrotizing soft-tissue infection. J. Investig. Med..

[26] Chung S.W., Liu X., Macias A.A., Baron R.M., Perrella M.A. (2008). Heme oxygenase-1-derived carbon monoxide enhances the host defense response to microbial sepsis in mice. J. Clin. Investig..

[27] Weiss S.J. (1989). Tissue destruction by neutrophils. N. Engl. J. Med..

[28] Wilgus T.A., Roy S., McDaniel J.C. (2013). Neutrophils and Wound Repair: Positive Actions and Negative Reactions. Adv. Wound Care.

[29] Baiula M., Greco R., Ferrazzano L., Caligiana A., Hoxha K., Bandini D., Longobardi P., Spampinato S., Tolomelli A. (2020). Integrin-mediated adhesive properties of neutrophils are reduced by hyperbaric oxygen therapy in patients with chronic non-healing wound. PLoS ONE.

[30] Ryter S.W. (2020). Therapeutic Potential of Heme Oxygenase-1 and Carbon Monoxide in Acute Organ Injury, Critical Illness, and Inflammatory Disorders. Antioxidants.

[31] Eltzschig H.K., Carmeliet P. (2011). Hypoxia and inflammation. N. Engl. J. Med..

[32] Babchin A., Levich E., Melamed M.Y., Sivashinsky G. (2011). Osmotic phenomena in application for hyperbaric oxygen treatment. Colloids Surf. B Biointerfaces.

[33] Barshtein G., Pajic-Lijakovic I., Gural A. (2021). Deformability of Stored Red Blood Cells. Front. Physiol..

[34] Steenebruggen F., Jacobs D., Delporte C., Van Antwerpen P., Boudjeltia K.Z., Biston P., Piagnerelli M. (2023). Hyperbaric oxygenation improve red blood cell deformability in patients with acute or chronic inflammation. Microvasc. Res..

[35] Stevens D.L. (2000). The pathogenesis of clostridial myonecrosis. Int. J. Med. Microbiol..

[36] Buboltz J.B., Murphy-Lavoie H.M. (2025). Gas Gangrene. [Updated 2023 Jan 30]. StatPearls [Internet].

[37] Bakker D.J. (2012). Clostridial myonecrosis (gas gangrene). Undersea Hyperb. Med..

[38] Titball R.W., Naylor C.E., Basak A.K. (1999). The Clostridium perfringensα-toxin. Anaerobe.

[39] O’Brien D.K., Melville S.B. (2004). Effects of Clostridium perfringens alpha-toxin (PLC) and perfringolysin O (PFO) on cytotoxicity to macrophages, on escape from the phagosomes of macrophages, and on persistence of C. perfringens in host tissues. Infect. Immun..

[40] Maclennan J.D. (1962). The histotoxic clostridial infections of man. Bacteriol. Rev..

[41] van Unnik A.J.M. (1965). Inhibition of toxin production in Clostridium perfringens in vitro by hyperbaric oxygen. Antonie Van Leeuwenhoek.

[42] Nora P.F., Bransfield J., Cieslak F., Laufman H. (1966). HPO in clostridial toxicity and strangulation obstruction. Arch. Surg..

[43] Hirn M. (1993). Hyperbaric oxygen in the treatment of gas gangrene and perineal necrotizing fasciitis. A clinical and experimental study. Eur. J. Surg. Suppl..

[44] Holland J.A., Hill G.B., Wolfe W.G., Osterhout S., Saltzman H.A., Brown I.W. (1975). Experimental and clinical experience with hyperbaric oxygen in the treatment of clostridial myonecrosis. Surgery.

[45] Wilkinson D., Doolette D. (2004). Hyperbaric oxygen treatment and survival from necrotizing soft tissue infection. Arch. Surg..

[46] Hedetoft M., Bennett M.H., Hyldegaard O. (2021). Adjunctive hyperbaric oxygen treatment for necrotising soft-tissue infections: A systematic review and meta-analysis. Diving Hyperb. Med..

[47] Shishido A., Schrank G., Vostal A., Uehling M., Tripathi R., Chintalapati S., Conway L., Kus N., DiChiacchio L., Kai M. (2025). Hyperbaric Oxygen Therapy for Necrotizing Soft Tissue Infections: A Retrospective Cohort Analysis of Clinical Outcomes. Surg. Infect..

[48] Anderson C.A., Jacoby I. (2023). Necrotizing soft tissue infections. Hyperbaric Medicine Indications Manual.

[49] Weening R.P., Giannakopoulos G.F., van Hulst R.A. (2023). Clostridial myonecrosis (gas gangrene). Hyperbaric Medicine Indications Manual.

[50] Sethuraman K.N., Smolin R., Henry S. (2022). Is There a Place for Hyperbaric Oxygen Therapy?. Adv. Surg..

[51] Schiavo S., Brenna C.T.A., Albertini L., Djaiani G., Marinov A., Katznelson R. (2024). Safety of hyperbaric oxygen therapy in patients with heart failure: A retrospective cohort study. PLoS ONE.

[52] Jokinen-Gordon H., Barry R.C., Watson B., Covington D.S. (2017). A Retrospective Analysis of Adverse Events in Hyperbaric Oxygen Therapy (2012–2015): Lessons Learned From 1.5 Million Treatments. Adv. Ski. Wound Care.

[53] Gengel K.C., Hendriksen S., Cooper J.S. (2025). Hyperbaric Related Myopia and Cataract Formation. [Updated 2023 Jul 4]. StatPearls [Internet].

[54] Sheffield P.J., Desautels D.A. (1997). Hyperbaric and hypobaric chamber fires: A 73-year analysis. Undersea Hyperb. Med..

[55] Carlson S., Jones J., Brown M., Hess C. (1992). Prevention of hyperbaric-associated middle ear barotrauma. Ann. Emerg. Med..

